# Correction: miR-449a Suppresses LDHA-Mediated Glycolysis to Enhance the Sensitivity of Non-Small Cell Lung Cancer Cells to Ionizing Radiation

**DOI:** 10.32604/or.2026.086717

**Published:** 2026-06-16

**Authors:** Liang Li, Huijuan Liu, Lianjiang Du, Pan Xi, Qian Wang, Yanqin Li, Di Liu

**Affiliations:** 1 Department of Radiotherapy, Shaanxi Provincial Tumor Hospital, Xi’an, China; 2 Department of Oncology, Ankang City Central Hospital, Ankang, China; 3 School of Public Health, Xi’an Jiaotong University Health Science Center, Xi’an, China; 4 Department of Oncology, The Second Affiliated Hospital of Xi’an Jiaotong University, Xi’an, China

In the article “miR-449a Suppresses LDHA-Mediated Glycolysis to Enhance the Sensitivity of Non-Small Cell Lung Cancer Cells to Ionizing Radiation” (Oncol Res, 2018, Vol. 26, No. 4, pp. 547–556. doi:10.3727/096504017X15016337254605), an inadvertent error occurred during the compilation of Fig. 3A and Fig. 3C. This needed corrections to ensure the accuracy and integrity of the data presented.

Issue with Fig. 3A:

• Original Issue: Following the publication of the above article, an interested reader drew to the authors’ attention that unexpected area of similarity in flow cytometry data in Fig. 3A on page 552 were identified. After having re-examined their original data, the authors realized that the wrong data had inadvertently been included in this figure during the image compilation.
• Reason for Change: To accurately represent the apoptosis of lung cancer cells in response to various treatments, we re-examined and analyzed the original data and revised Fig. 3A,B. The new images are correctly provided and the description of apoptotic fractions in the two cells under miR-449a mimics treatment were corrected accordingly in results text and now all information accurately corresponds to the correct experimental condition.
• Impact on Results: It replaces the erroneous images with the correct ones, ensuring that the visual data accurately corresponds to the reported experimental findings. This correction does not affect the results or conclusions of the article. The authors apologize for this oversight and appreciate the opportunity to correct the errors.

Issue with Fig. 3C:

• Original Issue: Concerns were raised by an interested reader that the bands of γ-H2AX under treatments of “IR, IR+NC and IR+mimics” in H1299 cells showed similar appearance with the western blot data presented in another published study. To make the results more accurate and clear, we replaced this panel of western blot data.
• Reason for Change: To accurately represent the γ-H2AX expression of H1299 cells in response to various treatments, the new images are correctly provided and now accurately corresponds to the correct experimental condition.
• Impact on Results: This correction does not affect the overall conclusion of the study.

A correction has been made in Section: Results, Sub-section: miR-449a Restoration Promotes IR-Induced Cell Apoptosis and DNA Damage. The corrected versions of Fig. 3 are provided. The changes were necessary to maintain the integrity of the published work and to provide accurate visual data to support the study’s findings. We confirm that these corrections do not alter any of the study’s results or conclusions, and we apologize for any inconvenience caused by the errors.

The authors would like to correct the figure as follows:


**Page. No.**

**Exact figure to be corrected**

**Correction**
552
Fig. 3
Replace with new Fig. 3

**Figure 3**

**miR-449a Restoration Promotes IR-Induced Cell Apoptosis and DNA Damage**

In addition, we evaluated cell apoptosis of lung cancer cells using the annexin V/PI method. The cancer cells were introduced with miR-449a mimics or the corresponding control and then were exposed to IR at 8 Gy. After 48 h of incubation, the fraction of apoptotic cells in A549 further increased to 31.7 ± 0.9% and to 34.9 ± 1.7% in H1299 cells (Fig. 3A,B). We determined the expression of γ-H2AX, a hallmark of DNA damage, using Western blot. We found that IR induced a significant increase in γ-H2AX expression compared to the non-IR- treated cells, which were profoundly corresponding by the administration of miR-449 mimics (Fig. 3C). Elevated γ-H2AX plaques were observed in IR-exposed A549 cells that were transfected with miR-449a mimics (Fig. 3D).

**Figure 3:**
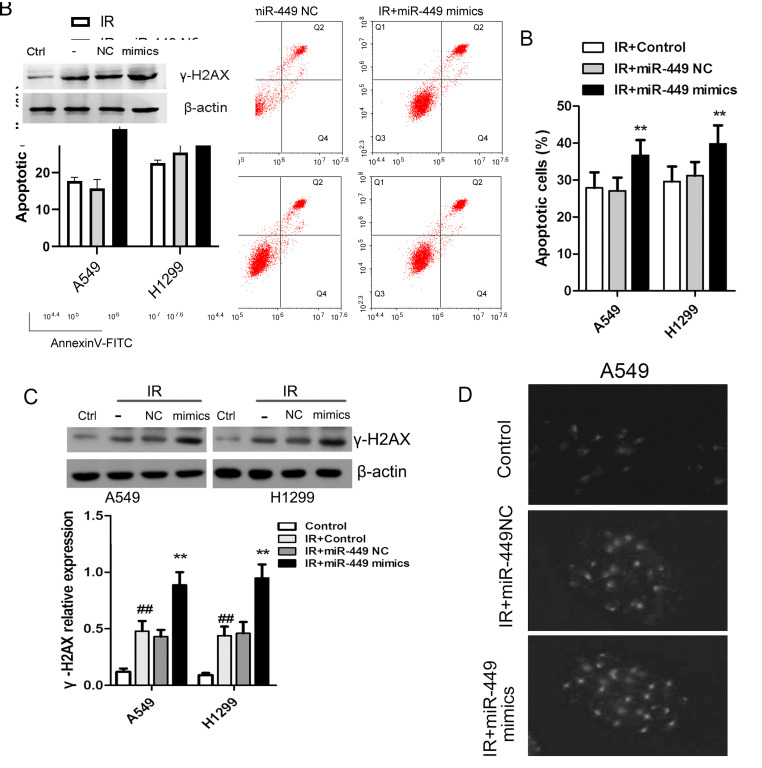
miR-449a upregulation enhanced IR-induced cell apoptosis and DNA damage in lung cancer cells. (**A**,**B**) Cell apoptosis in IR-exposed cells in the presence or absence of miR-449 mimics. (**C**) Western blot analysis of the level of DNA double-strand break marker (γ-H2AX) after IR. **p < 0.01, compared to IR + miR-449 NC; ^##^*p* < 0.01, compared to Control. (**D**) After irradiation, A549 cells were fixed and stained with anti-γ-H2AX antibodies.

